# Night eating syndrome prevalence and its association with sleep quality, eating patterns, and psychopathology in an Israeli community sample

**DOI:** 10.1007/s00127-025-02880-w

**Published:** 2025-04-16

**Authors:** Orna Tzischinsky, Yael Latzer

**Affiliations:** 1 Department of Behavioral Sciences, Emek Yezreel Academic College, Emek Yezreel, Israel; 2 Eating disorders institution, Psychiatric division, Rambam Health Care campus, Haifa, Israel; 3 Faculty of Social Welfare and Health Studies, University of Haifa, 199, Abba Khoushy av, Haifa, 3103301 Israel

**Keywords:** Sleep disturbances, Night eating syndrome, Prevalence, Community sample, Eating pattern, Psychopathology

## Abstract

**Introduction:**

Night eating syndrome (NES) involves hyperphagia and nocturnal ingestion, causing significant distress and impairment. Despite its impact, NES is poorly understood and underdiagnosed both in clinical and community settings. Prevalence rates vary, highlighting the need for further research in community samples.

**Purpose:**

To assess NES prevalence in a community sample and its relationship with sleep disturbances, eating patterns, and psychopathology.

**Method:**

A total of 746 participants (ages 21–51), including 377 men (50.5%), were recruited through a large Israeli online platform. Participants completed self-report demographic data and questionnaires assessing NES, sleep disturbances, eating patterns, and psychopathology.

**Results:**

The prevalence of NES (night eating questionnaire/NEQ: score > 25, score > 21) was 8.8% and 18.2%, respectively. No significant differences in NES prevalence were found between genders or age groups in most of the variables. There were no significant differences between the NES and non-NES groups in terms of BMI, age, or gender. However, significant differences were found in sleep disturbances (PSQI total), depression, and anxiety. The NES group was significantly associated with higher levels of fat and carbohydrate consumption during the evening and night.

**Conclusion:**

The NES prevalence among study participants was relatively higher than among previous community samples worldwide, despite participants having a BMI within the normal range. The higher prevalence, along with the significant associations with lower sleep quality, higher levels of anxiety and depression, and increased fat and carbohydrate consumption, underscore the need for greater emphasis on the diagnosis, treatment, and prevention of NES in the community beyond cultural differences.

## Introduction

Night eating syndrome (NES) is characterized by a delayed circadian pattern of food intake, with at least 25% of daily energy consumed after dinner (evening hyperphagia) and/or at least two episodes of nocturnal eating per week after sleep onset (nocturnal ingestion) (Criterion A). Night eating syndrome is differentiated from sleep-related eating disorders via the awareness and recall of nocturnal eating episodes. Additionally, the daily pattern of NES is manifested by at least three of the following features (Criterion C): morning anorexia; a strong urge to eat between dinner and sleep onset, and/or during the night; insomnia; a belief that one must eat in order to sleep; and worsening of depression in the evening. The disorder is associated with significant distress (Criterion D), lasts for at least three months, and is not secondary to a medical or psychiatric disorder (Criterion E) [1]. These proposed criteria served as the basis for the most updated reference for diagnosing NES and were adopted by the DSM-5 [2].

Night eating syndrome is associated with weight gain as well as psychological and physical comorbidities [3, 4]. Various studies have reported diverse prevalence rates of NES across different populations. Among individuals with obesity, it has been found to range from 6 to 16% [5, 6], and among individuals with psychiatric comorbidity, particularly depression, the NES prevalence was found to be 15% [7]. The prevalence of NES in the general population remains somewhat elusive, with estimates ranging from 1 to 4.6% [8]. In Korea, NES prevalence was found to be 0.6% [9]; 1.3% among young adults in Switzerland [10]; 1.5% in the general population in Arab countries [11]; 1.1% in Germany [12]; and 3% among urban female adults in India [13].

Additionally, NES was found to be more prevalent among medical students in Saudi Arabia at 10.3%, with a high prevalence of obesity and overweight, similar to what has been reported in the Saudi population; however, these results were not associated with BMI [14]. In Pakistan, 49.4% reported NES, and 27.9% reported depression, with a significant correlation found between NES and depression. Depression has been identified as a predictor of NES [15].

The prevalence of NES among general university students in other countries revealed rates of 4.2% in the United States [16], 7.3% in Saudi Arabia [17]; 5.8% in Egypt [18]; 1.6% in China [19], 23.4% in Malaysia [20]; and 9.5% in Turkey [21]. Notably, all these prevalence rates exceeded the night eating questionnaire (NEQ) threshold of > 25.

Night Eating Syndrome (NES) prevalence varies widely across populations and is influenced by obesity, psychiatric conditions, and demographic and cultural factors.

Among individuals with obesity, it ranges from 6 to 16%, while in the general population, estimates range from 0.6 to 4.6%. Among university students in Arab countries, prevalence rates range from 5.8 to 7.3%, compared to 1.6–23.4% in Western and Asian countries. Among medical students, NES prevalence varies significantly, ranging from 10.3 to 49.4%, with a notable association with depression in some populations. Across studies, NES prevalence exceeded the Night Eating Questionnaire (NEQ) threshold (> 25).

These varying rates across different studies underscore the need for further research to comprehensively understand the multifaceted factors contributing to NES in the community. Differences in NES prevalence rates may be attributed to various factors and mechanisms, including sleep and eating patterns, as well as demographic variables such as gender and age range.

Regarding the sleep factor, NES has been found to be associated with certain neuroendocrine disorders, including decreased plasma concentrations of leptin and melatonin at night [22, 23]. This finding suggests that individuals with NES may consume more food at night due to lower levels of leptin, a hormone that regulates hunger. Additionally, they may experience difficulty sleeping due to reduced plasma levels of melatonin, a hormone that regulates sleep [24]. These alterations in the hypothalamic-pituitary-adrenal axis, which is crucial for regulating sleep and wake cycles, may contribute to disrupted sleep patterns in individuals with NES [25].

Via the use of self-report questionnaires and objective measures such as actigraphy, recent studies have found correlations between poor sleep quality and NES [26, 27]. In addition, a high correlation was found between emotional eating, NES, and sleep disturbances. Emotional eaters demonstrate a higher likelihood (6.5 times) of having NES compared to those who do not engage in emotional eating [28]. Individuals experiencing negative emotions before sleep may wake up at night and turn to eating as a means of self-soothing and improving sleep quality. Moreover, call center employees who experience high distress and low depression before bedtime have been found to wake up more during the night and engage in emotional eating [29].

Regarding eating factors, despite being a core criterion for NES, the eating patterns of individuals with NES have received relatively little research attention. Studies have revealed that individuals with NES, particularly in clinical samples, tend to have a higher energy intake after the evening meal before sleep, and during nighttime after sleep onset, and they frequently skip breakfast compared to individuals without NES [30, 31]. Further research has compared dietary patterns, energy intake, and macronutrient composition between individuals with binge eating behavior with and without NES, revealing significantly higher frequency of binge days and episodes, higher energy intake and fat consumption, and lower protein consumption among those with both binge eating behavior and NES compared to those with binge eating behavior without NES [32].

In community samples, findings regarding energy intake differences between individuals with NES and controls have been inconsistent. Some studies have shown higher energy intake among individuals with NES compared to controls, whereas in other studies, no significant differences were found [33, 34]. Additionally, community studies among U.S. college students and Korean adolescents reported no significant differences in eating habits between students with or without NES [35, 36]. Th**e** underlying mechanisms that may explain the relationships between NES to emotional eating, stress, and psychiatric disorders may be explained by how dysregulation of the hypothalamic-pituitary-adrenal (HPA) axis leads to abnormal cortisol secretion patterns, which in turn influences stress-related eating behaviors. Individuals with NES often experience heightened stress responses, which may increase cravings for high-calorie foods, particularly at night. Additionally, another mechanism can be related to this relationship, like how circadian rhythm disturbances can disrupt appetite regulation and contribute to nighttime eating tendencies, particularly in individuals with psychiatric comorbidities such as depression [37, 38].

To the best of our knowledge, NES prevalence in the general population is inconsistent, with the highest rates observed mainly among people with high psychiatric comorbidity and/or populations with obesity [33, 39]. Night eating syndrome has been found to be associated with poor sleep quality, which, in turn, has been linked with obesity, delayed calorie intake, and poor eating habits [40–43]. The relationship between these factors in the general population and their connection to sleep disturbances and eating patterns remains unclear.

Therefore, this cross-sectional study was conducted to estimate NES prevalence and NES association with sleep disturbances and eating patterns among a community sample in the Israeli population. The aims of the current study were:

1. To assess NES prevalence in a community sample, taking demographic factors into consideration.
2. To examine the relationship between NES and various factors related to sleep, depression, anxiety, and body mass index (BMI).
3. To assess the level of sleep disturbances, psychiatric disorders, depression, anxiety, BMI, and eating patterns (evening-time and night-time) among individuals with NES vs. those without NES (non-NES) in a community sample.

## Method

### Participants

Study participants included 746 individuals, comprising 377 (50.5%) men (mean age 35.8 ± 7.9 years, range 21–51 years) and 369 (49.5%) women (mean age 35.75 ± 7.84, range 21–50 years). Inclusion criteria: Israeli Hebrew-speaking individuals aged 21 and up.: Participants were recruited in August 2023 through iPanel (https://www.iPanel.co.il), a large Israeli online platform with over 100,000 panel members from the community. iPanel participants complete tasks for points, which can be converted into gift certificates.

### Measures

The data, including demographic, clinical, sleep quality, night eating (including evening hyperphagia and night ingestion), and eating patterns, were collected using self-administered questionnaires (via the Qualtrics platform). The clinical questionnaire assessed participants’ substance use, including drug, cigarette, and alcohol consumption, as well as any history of psychiatric disorder diagnoses. The sociodemographic questionnaire included weight, height, age, gender, marital status, education level, and working status.

The eating patterns questionnaire collected information on energy intake and macronutrient composition during the evening after dinner and after sleep onset. Macronutrient consumption included carbohydrates, fat, protein, vegetables, and fruits.

Night eating questionnaire (NEQ). The behavioral and psychological characteristics of NES were assessed using the NEQ [44]. The NEQ consists of 14 questions which cover morning appetite, time of the first meal, food cravings, control overeating habits both before going to sleep and during night awakenings, food consumption after dinner, initial insomnia, frequency of nocturnal awakenings and food intake during these awakenings, depressive symptoms, and awareness of nighttime eating episodes. Responses are rated on a Likert scale from 0 to 4, with 0 indicating no NES and 4 indicating a high likelihood of NES (three questions are reverse scored). The total NEQ score ranges from 0 to 52 points. A score of 25 or above is used as a threshold for diagnosing NES [44], and a score of 21 or above may also be used as a cutoff value [45]. The Cronbach’s alpha for the NEQ tool is 0.70 [44, 45]. In the current study, it was found to be 0.59.

Sleep quality: The Pittsburgh Sleep Quality Index (PSQI) [46]; Hebrew version [47] is a self-administered tool designed to assess sleep quality in both clinical and general populations. The PSQI includes 18 questions: 14 are rated on a scale from 0 to 3, and four are open-ended questions intended to assess perceived sleep quality. These items generate categorical scores for the PSQI’s seven subcomponents and an overall score, representing the total subcomponents. The overall score provides a comprehensive summary of the participants’ sleep experiences and quality over the past two weeks, with higher scores indicating worse sleep quality. A total score above 5 suggests the presence of sleep disturbances. The Cronbach’s alpha for the PSQI tool in the clinical sample was 0.7, and it was found to be 0.72 in this study.

Anxiety: The Generalized Anxiety Disorder 2-item (GAD-2; [48, 49] Hebrew version: [50] is a concise screening instrument used to detect potential cases of GAD, based on the extended GAD-7. It comprises two items that evaluate the frequency of anxiety symptoms over the preceding two weeks. Each item is rated from 0 to 3, resulting in a total score range of 0 to 6. A score of 3 or higher is considered a positive screen for GAD, suggesting the need for further assessment. The Cronbach’s alpha for the GAD-2 questionnaire was approximately 0.82. In the current study, the Cronbach’s alpha for the anxiety instrument was 0.81.

Depression: The Patient Health Questionnaire-2 (PHQ-2; [51]) Hebrew version: [52] is a brief screening tool used to identify likely cases of major depressive disorder based on the longer PHQ-9. It includes two questions that measure the frequency of depression and anhedonia (loss of interest or pleasure) symptoms over the past two weeks. Each item is rated from 0 to 3, yielding a total score range of 0 to 6. A score of 3 or more is considered a positive screen for depression, indicating that further evaluation might be necessary. The Cronbach’s alpha for the PHQ-9 questionnaire was 0.83. In the present study, the Cronbach’s alpha for the PHQ-2 questionnaire was 0.70.

### Statistical analyses

All statistical analyses were performed using IBM SPSS version 27. The results of this study are presented as frequencies and proportions for categorical variables and as means and standard deviations for continuous variables. The chi-square test (χ²) was employed to assess the relationship between categorical variables and NES, whereas an independent t-test or Mann-Whitney test, in the case of non-normally distributed data, was used to examine the association of continuous variables with NES. The cut-off points used to measure NES prevalence were 21 and above, according to Latzer et al. (2014) [45], and 25 and above, according to [44].

Pearson correlation was utilized to evaluate the relationship between NEQ scores, age, BMI, PSQI-total, depression, and anxiety. A stepwise regression analysis was conducted, with age and gender forced into the model, to identify predictors of NES.

The average number of types of carbohydrates, fats, and proteins consumed in the evening and nighttime was calculated for all participants. The chi-square test (χ²) was used to compare individual food items between individuals with and without NES, whereas the independent t-test was employed to compare the mean number of these items between NES and non-NES participants. The McNemar test was applied to determine whether there were differences in evening and nighttime consumption patterns of carbohydrates, fats, and proteins.

Given that most studies have used a cut-off score of > 25 [44], with only one study indicating a cut-off score of > 21 for NES diagnosis [45], we decided to report NES prevalence in the general population using both cut-offs. However, for the remainder of the analysis, we used the cut-off of > 25, as done by most researchers.

### Ethical approval

#### Ethical approval

was obtained from the Emek Yezreel College Ethics Committee (Reference No: YVC Emek 2023-75) prior to data collection. All participants provided written consent to participate. Privacy and confidentiality of all collected data were maintained throughout the study.

## Results

Demographic, NES (NEQ-total), anxiety, depression, and sleep disturbances (PSQI-total) variables revealed no significant gender differences in age, BMI, marital status, employment status, PSQI-total, or NEQ-total and depression scores. However, significant differences were observed in education levels and anxiety, with education and anxiety being higher in women than in men (see Table 1).

**Table 1:** Descriptive statistics of demographic, PSQI-total, NEQ-total, depression, and anxiety data by gender

Variable	All (M ± SD)	Women (M ± SD)	Men (M ± SD)	Significance
Age	35.77 ± 7.88 (21–51)	35.75 ± 7.84	35.79 ± 7.93	T = 0.07 (df = 744); n.s.
BMI	25.94 ± 4.76 (17.91–42.21)	25.62 ± 5.29	26.22 ± 4.22	T = 1.87 (df = 741); n.s.
Education	14.44 ± 2.79	14.74 ± 2.57	14.14 ± 3.0	T = 2.9 (df = 741); *p* < 0.05
Marital Status: In relation (%)	71.8	71.3	72.4	χ²=0.12; n.s.
Marital Status: Separated (%)	28.2	28.7	27.6	χ²=0.12; n.s.
Employed: Yes (%)	89.1	88.1	90.2	χ²=0.86; n.s.
Employed: No (%)	10.9	11.9	9.8	χ²=0.86; n.s.
Depression	3.59 ± 1.50	3.51 ± 1.45	3.67 ± 1.54	T = 1.38, (df = 691); n.s.
Anxiety	3.79 ± 1.56	3.99 ± 1.57	3.60 ± 1.53	T = 3.26, (df = 691); *p* < 0.001
PSQI-total	7.20 ± 2.75	7.26 ± 2.66	7.13 ± 2.83	T = 0.63 (df = 687); n.s.
NEQ-total	15.42 ± 6.22	15.43 ± 6.02	15.4 ± 6.43	T = 0.07 (df = 744); n.s.

There were significant positive correlations observed between NEQ-total and PSQI-total (*r* = 0.42, *p* < 0.001), depression (*r* = 0.38, *p* < 0.001), and anxiety (*r* = 0.36, *p* < 0.001), suggesting that higher NEQ-total scores are linked with more severe PSQI-total, higher levels of depression, and increased anxiety. No significant correlation was identified between NEQ-total and BMI (see Table 2).

**Table 2:** The correlation between BMI, sleep disturbances (PSQI-total), NEQ-total, depression, and anxiety

Variable	PSQI-total	BMI	Depression	Anxiety
NEQ-total	0.42**	0.02	0.38**	0.36**
PSQI-total		0.06	0.41**	0.41**
BMI	0.06		0.03	0.03
Depression	0.41**	0.03		0.70**

** The “**” indicates statistical significance *p* < 0.001

After adjusting for age and gender, PSQI-total (*p* < 0.001), depression (*p* < 0.001), and anxiety (*p* = 0.03) were significant predictors of NEQ-total, although anxiety explained only 0.5% of the variance. For every 1-point increase in PSQI-total, NEQ-total increased by 0.77 (b = 0.768, se: 0.095; 95% CI: 0.58-0.96), and for every 1-point increase in depression, NEQ-total increased by 0.72 (b = 0.723, se: 0.202; 95% CI: 0.33-1.12) (see Table 3).

**Table 3:** Regression analysis of NEQ-total scores and their association with PSQI-total, depression, and anxiety levels

Variable	B	Std. Error	Beta	T	Sig.	Lower Bound	Upper Bound	R2 change
Gender	-0.43	0.427	-0.035	-1.008	0.314	-1.268	0.408	
Age	-0.048	0.027	-0.06	-1.746	0.081	-0.101	0.006	0.001
PSQI-total	0.768	0.095	0.31	8.07	< 0.001	0.581	0.955	0.18
Depression	0.723	0.202	0.174	3.58	< 0.001	0.327	1.12	0.048
Anxiety	0.425	0.195	0.107	2.177	0.03	0.042	0.808	0.005

Furthermore, we compared the characteristics of the NES group (NEQ > 25) with those of the non-NES group. The results indicated that there were no significant differences between the NES and non-NES groups in terms of age, gender, BMI, drug use, or alcohol consumption. However, the NES group had significantly lower education levels (mean of 13.73 years) compared to the non-NES group (mean of 14.51 years) and were less likely to be employed (7.5% vs. 92.5%). Regarding sleep disturbances, the NES group exhibited a higher mean PSQI-total score and reported more instances of sleep apnea, somnambulism, and restless legs syndrome. The NES group also had a higher prevalence of psychiatric disorders, particularly higher levels of anxiety and depression, compared to the non-NES group. Additionally, cigarette smoking was significantly more prevalent among participants with NES compared to those without NES (see Table 4).

**Table 4:** Comparison of demographics, sleep disturbances, BMI, substance use, depression, anxiety, and psychiatric disturbances between NES and non-NES according to cutoff 25 in NEQ-total

Variable	NES (*N* = 66, 8.8%)	Non-NES (*N* = 680, 91.2%)	Significance
Age	34.89 ± 7.8	35.85 ± 7.88	T = 0.94, df = 741; n.s.
Education	13.73 ± 2.52	14.51 ± 2.8	T = 2.38, df = 744, *p* = 0.01
BMI	25.96 ± 5.62	25.86 ± 5.31	T = 0.12, df = 692, n.s.
PSQI-total	± 3.59.57	± 2.556.97	t = 7.45, df = 716, *p* > 0.001
Gender: men	9.8%	90.2%	χ²=0.88, n.s.
Gender: women	7.9%	92.1%	χ²=0.88, n.s.
Employed: Yes	7.5%	92.5%	χ²=13.4, *p* = 0.001
Employed: No	19.8%	80.2%	χ²=13.4, *p* = 0.001
Sleep Apnea (%)	9.1	3.4	χ²=5.3, *P* = 0.02
Somnambulism (%)	9.1	3.2	χ²=5.3, *P* = 0.02
Restless legs syndrome (%)	30.3	11.3	χ²=19.2, *P* > 0.001
Psychiatric Disorders (%)	16.7	5.1	χ²=13.8, *P* > 0.001
Depression	4.54 ± 1.62	3.50 ± 1.45	T = 5.39, df = 691, *p* < 0.001
Anxiety	4.92 ± 1.77	3.68 ± 1.49	T = 6.17, df = 691, *P* < 0.001
Drug use (%)	12.1	6.8	χ²=2.6, n.s.
Cigarette use (%)	47	26.3	χ²=12.7, *P* > 0.001
Alcohol use (%)	37.9	29.7	χ²=1.9, n.s.

Regarding eating patterns, comparisons were made between the NES and non-NES groups for evening and nighttime eating behaviors.

### Evening-time eating

The NES group exhibited a significantly higher rate of evening eating across all nutrient categories, except for fruits and vegetables. Specifically, during the evening, 56.1% of the NES group consumed carbohydrates, 74.2% consumed fats, and 63.6% consumed proteins. In contrast, in the non-NES group, 30.6% consumed carbohydrates, 55.1% consumed fats, and 42.9% consumed proteins. Among participants who consumed carbohydrates, there was no difference in the average number of carbohydrate types (categories) consumed between the groups. Similarly, no difference was found in the mean number of protein types (categories) consumed by those who ate proteins. However, there was a statistically significant difference in the mean number of fat types (categories) consumed between the groups (NES median = 2 vs. non-NES median = 1) (see Table 5).

### Nighttime eating

The NES group also had a significantly higher rate of nighttime eating for all nutrient categories, except for meat consumption. During the nighttime, 31.8% of the NES group consumed carbohydrates, 60.6% consumed fats, and 31.7% consumed proteins, compared to 4.0% of the non-NES group for carbohydrates, 9.1% for fats, and 4.0% for proteins. There were no statistically significant differences between the two groups regarding the distribution of carbohydrate, fat, or protein consumption among those who consumed these nutrients.

### Evening-time eating vs. nighttime eating

Of the 37 participants who consumed carbohydrates during the evening, 16 (43.2%) also consumed them at night. Among the 29 NES participants who did not consume carbohydrates in the evening, 4 (13.8%) did so at night. The pattern of carbohydrate consumption differed significantly between evening and nighttime eating (McNemar *p* < 0.001). Among the 49 NES participants who consumed fats during the evening, 37 (75.6%) also consumed them at night. Of the 17 NES participants who did not consume fats in the evening, 3 (17.6%) consumed them at night. The difference in fat consumption between evening and nighttime eating was statistically significant (McNemar *p* = 0.035). For proteins, 19 of the 42 NES participants who consumed them during the evening (45.2%) also consumed them at night, whereas 2 of the 24 NES participants who did not consume proteins in the evening (8.3%) consumed them at night. The difference in protein consumption between evening and nighttime eating was statistically significant (McNemar *p* < 0.001) (see Table 5).

**Table 5:** Comparison between NES-group vs. non-NES in eating patterns in the evening-time and at night-time

Variable	Evening-time eating NES (*N* = 66)	Evening-time eating Non-NES (*N* = 680)	Evening-time *p*-value	Night-time eating NES (*N* = 66)	Night-time eating Non-NES (*N* = 680)	Night-time *p*-value
Bread	23 (34.8)	109 (16.0)	< 0.001	9 (13.6)	12 (1.8)	< 0.001
Potato/rice	20 (30.2)	82 (12.1)	< 0.001	5 (7.6)	7 (1.0)	0.002
Pasta	15 (22.7)	70 (10.3)	0.002	4 (6.1)	6 (0.9)	< 0.001
Grains	17 (25.8)	106 (15.6)	0.03	10 (15.2)	14 (2.1)	< 0.001
Mean number of types	0.28 ± 0.33	0.13 ± 0.25	< 0.001	0.11 ± 0.19	0.01 ± 0.08	< 0.001
Peanut butter	13 (19.7)	42 (6.2)	< 0.001	8 (12.1)	5 (0.7)	< 0.001
Sweets	44 (66.7)	335 (49.3)	0.007	29 (43.9)	51 (7.5)	< 0.001
Chips	28 (42.8)	195 (28.7)	0.02	18 (27.3)	23 (3.4)	< 0.001
Mean number of types	0.58 ± 0.22	0.51 ± 0.20	0.008	0.47 ± 0.17	0.40 ± 0.13	0.02
Meat	12 (18.2)	62 (9.1)	0.02	1 (1.5)	6 (0.9)	0.48
Ice cream	40 (60.6)	272 (40.0)	0.001	20 (30.3)	24 (3.5)	< 0.001
Mean number of types	0.62 ± 0.22	0.57 ± 0.17	0.17	0.50 ± 0.00	0.56 ± 0.16	0.08
Fruits and vegetables	29 (43.9)	242 (35.6)	0.18	11 (16.7)	27 (4.0)	< 0.001

## Discussion

The aim of this cross-sectional study was to estimate NES prevalence and the association of NES with sleep disturbances and eating patterns in a community sample from Israel.

The findings showed that NES prevalence was 18.2% based on a cutoff score of 21 on the NEQ and 8.8% based on a cutoff score of > 25, indicating that NES is relatively common in Israel’s general population. The literature supports two cutoff points for diagnosing NES, with most studies using 25 or above and one study suggesting 21 or above. Given that most studies have used a cut-off score of > 25 [44], with only one study indicating a cut-off score of > 21 for NES diagnosis [45], we decided to report NES prevalence in the general population using both cut-offs. However, for the remainder of the analysis, we used the cut-off of > 25, as done by most researchers. Using a cutoff of 25 or above, NES prevalence in Israel appears high compared to other countries.

Our results are consistent with studies of clinical samples, showing a high prevalence of NES among individuals with obesity (6-16%) and psychiatric comorbidities, particularly depression (15%). Similar prevalence rates have been reported in Turkey (9.5%) and Malaysia (23.4%). However, our findings differ from studies reporting lower NES prevalence in general populations, where NES prevalence has been reported to be substantially low, typically ranging from 1 to 4.6% across different countries. This discrepancy may be attributed to methodological differences, including variations in sampling methods, diagnostic criteria, and cultural factors influencing eating and sleeping behaviors. Regarding cultural differences, the higher NES rate in Israel may reflect ongoing existential tension, stress, and trauma, particularly in the last five years due to the COVID-19 pandemic and increased terror attacks [53, 54, 55]. These findings add to the literature by providing an updated NES prevalence in community samples and highlighting the relationship between NES and stress, as seen in studies of medical students in Pakistan (49.36%) and call center workers in Malaysia (12.0%).

The current results show no significant relationship between obesity levels, mean BMI, and NEQ levels, nor significant differences between NES and non-NES groups. The research population was within the normal BMI range. These findings contrast with earlier studies linking BMI with NES severity, especially in clinical samples and among individuals seeking weight loss treatment [5, 6]. However, they align with one study conducted among a community sample, showing no relationship between NES and BMI [14]. These findings might be due to the lower psychopathology typically found in community versus clinical samples. One possible explanation for this discrepancy may lie in the differences between clinical and community samples in terms of psychopathology, eating behaviors, and weight-related concerns. Individuals in clinical settings, particularly those seeking treatment for obesity or eating disorders, often exhibit higher psychological distress, greater emotional eating tendencies, and increased concerns about weight control, which may intensify the relationship between NES and BMI. In contrast, community samples typically include individuals with more varied and milder eating behaviors, as well as lower levels of emotional distress, which might weaken or obscure this association. Additionally, selection bias in clinical samples—where individuals experiencing greater impairment are more likely to seek treatment—may further explain the stronger association between NES and BMI observed in those settings.

The current results also found no significant gender differences in NES prevalence, sleep disturbances, depression, or NEQ total scores, suggesting NES affects both genders similarly in the general population. Notably, women had slightly higher education levels, and anxiety was significantly higher in women than in men. These results are consistent with previous research indicating higher anxiety rates among females, in general, including Israel [53].

These results imply that gender and age do not significantly influence NES prevalence in the general population, contrasting with clinical findings where NES was found to be more prevalent among women [56]. This difference could be related to BMI, as NES is more common among individuals with obesity (6-16%) [54, 57] than among the general population (1.5%) [58]. Given that the participants in our study had normal BMIs, gender differences might not be apparent.

Additionally, women’s higher help-seeking behaviors, driven by greater concern about weight and stress, may contribute to observed differences in clinical settings. Women are more likely to experience severe stress symptoms, leading to higher rates of anxiety and depression, which impacts overall well-being [59].

Regarding sleep disturbances, a strong association with NES was found. Individuals with NES had significantly higher PSQI-total scores, indicating poorer sleep quality. As NES severity increases, sleep disturbances also seem to increase.

Moreover, in the current study, NES was linked to higher rates of sleep apnea, somnambulism, and restless legs syndrome, which further disrupt sleep and may worsen the cycle of poor sleep and night eating. These findings align with previous research linking NES to disrupted sleep patterns and poorer sleep quality [26, 27]. Individuals with NES tend to have later sleep onset due to evening hyperphagia and frequent night ingestion, leading to insufficient and poorer quality sleep [27, 60]. Reduced plasma levels of melatonin and leptin in NES sufferers may also contribute to these disrupted sleep patterns and increased night ingestion [22–24].

The current results also found a higher prevalence of psychiatric comorbidities, particularly depression and anxiety, among individuals with NES. Depression and sleep quality were found to be predictors of NES severity. The significant correlation between NES, sleep disturbances, depression, and anxiety is consistent with the literature [30]. Additionally, NES is often comorbid with other eating disorders such as binge eating disorder and bulimia nervosa, with overlapping symptoms and distress [27, 59].

Regarding eating patterns, in the current study, NES sufferers consumed more nutrients, particularly carbohydrates, fats, and proteins, in the evening and at night compared to individuals who did not have NES. Participants with NES consumed more nutrients in the evening than during the night. These findings are consistent with studies among clinical samples that reported higher energy intake and fat consumption in NES sufferers [32, 33]. These findings may due also to the link of NES with dysregulation of the circadian system, particularly alterations in the timing of appetite and hormone secretion (e.g., melatonin, leptin, ghrelin) [37]. Additionally, the increased intake of carbohydrates, fats, and proteins in the evening and nighttime may link to metabolic consequences, such as insulin resistance and weight gain, further complicating health outcomes for individuals with NES. The comparison of evening and nighttime nutrient intake in a community sample is novel.

The increased food consumption in the NES group suggests that NES may serve as an emotion regulation mechanism, managing stress and emotional challenges. The higher evening food intake might be linked to traumatic life events, social isolation, or loneliness in the evening hours, where additional nighttime eating is needed for emotional control. Sleep disturbances may also trigger nocturnal eating as a form of self-soothing.

These explanations are supported by findings linking NES to emotional eating, where negative emotions before sleep trigger nocturnal eating episodes [28, 61]. These results highlight the importance of balanced nutrient treatment in managing NES in both clinical and community settings.

### Research limitations

Although the current study provides valuable insights into the prevalence and sleep/eating correlates of NES in the Hebrew-speaking general population in Israel, several limitations should be noted: First, the study’s cross-sectional nature limits the ability to establish causal relationships between NES and the associated factors. Second, the reliance on self-reported questionnaires, particularly through social media, may have introduced response biases, such as social desirability bias or recall bias, which could affect the accuracy of the reported information. Third, although the sample was community-based, it may not fully represent the broader Israeli population. Since the proportion of participants with NES was low, it was not possible to analyze subgroups within Israeli society, like immigrants from the former Soviet Union, Ethiopian Jews, Ultra-Orthodox Jews. Fourth, another limitation is that the internal reliability of the NEQ, PSQI, and PHQ-2 questionnaires is slightly low for subjective measurement. However, this reliability is around the same magnitude that found in other studies using these questionnaires [62–64]. Nevertheless, this remains a limitation that should be acknowledged. The fifth limitation highlights that, while comparing evening and nighttime nutrient intake in a community sample is novel, the breakdown of findings resulted in small sample sizes. The last limitation related to a potentially skewed sample, as education and employment status between the NES group vs. the NES group was found to be significantly different. Future studies should aim for a more diverse and representative sample to enhance generalizability. Forth, the use of different cutoff scores (21 and 25) for defining NES may have influenced the prevalence rates and associated findings. Standardization of cutoff scores is necessary for consistent comparisons across studies. Fifth, potential confounding variables, such as physical activity, dietary habits, ethnicity, and socioeconomic status, were not accounted for in this study. Future research should include these variables to better understand their impact on NES. Sixth, the study did not include objective measures of sleep disturbances (e.g., polysomnography or actigraphy) or eating behaviors (e.g., food diaries), which could have provided more accurate and detailed assessments [65–67].

## Conclusion, implications, and further research

This cross-sectional study estimated NES prevalence and NES association with sleep disturbances and eating patterns in an Israeli community sample. The prevalence of NES was 8.8% on the NEQ with a cutoff score of 25, indicating NES is relatively common in Israel, possibly due to unique stressors [53, 54, 68]. No significant relationship was found between obesity and NES severity, nor were gender differences in NES prevalence. A strong association was found between NES and poor sleep quality, with higher levels of depression and anxiety predicting NES severity. Individuals with NES also consumed more nutrients in the evening and at night than non-NES individuals. The study highlights cultural differences in NES prevalence, emphasizing the need for culturally tailored interventions. The varying prevalence rates across countries underscore the importance of cultural context in diagnosing and treating NES [9–11].

The study supports the existing literature on NES while providing new insights. By emphasizing the connection between eating behaviors, sleep patterns, and emotional states, the findings enhance our understanding of NES and its health implications.

Interventional studies focusing on improving sleep quality, healthy eating, and managing depression and anxiety could benefit NES treatment. Addressing NES in community samples is crucial to prevent deterioration that may lead to obesity and higher psychopathology. Future research should use longitudinal designs and objective measures to comprehensively understand NES and its implications.

## Data Availability

No datasets were generated or analysed during the current study.
